# Inclusion of medication-related fall risk in fall risk assessment tool in geriatric care units

**DOI:** 10.1186/s12877-020-01845-9

**Published:** 2020-11-06

**Authors:** Jana Michalcova, Karel Vasut, Marja Airaksinen, Katarina Bielakova

**Affiliations:** 1 Faculty of Pharmacy, Department of Applied Pharmacy, Masaryk University, Palackeho 1946/1, 612 42 Brno, Czech Republic; 2 Faculty of Pharmacy, Division of Pharmacology and Pharmacotherapy, Clinical Pharmacy Group, University of Helsinki, Viikinkaari 5E, 00014 Helsinki, Finland; 3 Clinic of Internal Medicine, Geriatrics and Practical Medicine, University Hospital Brno, Jihlavska 20, 625 00 Brno, Czech Republic

**Keywords:** Older adults, Fall risk, Medication, Hospital, Nursing home, Preventive risk management

## Abstract

**Background:**

Falls are common undesirable events for older adults in institutions. Even though the patient’s fall risk may be scored on admission, the medication-induced fall risk may be ignored. This study developed a preliminary categorization of fall-risk-increasing drugs (FRIDs) to be added as a risk factor to the existing fall risk assessment tool routinely used in geriatric care units.

**Methods:**

Medication use data of older adults who had experienced at least one fall during a hospital ward or a nursing home stay within a 2-year study period were retrospectively collected from patient records. Medicines used were classified into three risk categories (high, moderate and none) according to the fall risk information in statutory summaries of product characteristics (SmPCs). The fall risk categorization incorporated the relative frequency of such adverse drug effects (ADEs) in SmPCs that were known to be connected to fall risk (sedation, orthostatic hypotension, syncope, dizziness, drowsiness, changes in blood pressure or impaired balance). Also, distribution of fall risk scores assessed on admission without considering medications was counted.

**Results:**

The fall-experienced patients (*n* = 188, 128 from the hospital and 60 from nursing home records) used altogether 1748 medicaments, including 216 different active substances. Of the active substances, 102 (47%) were categorized as high risk (category A) for increasing fall risk. Fall-experienced patients (*n* = 188) received a mean of 3.8 category A medicines (*n* = 710), 53% (*n* = 375) of which affected the nervous and 40% (*n* = 281) the cardiovascular system. Without considering medication-related fall risk, 53% (*n* = 100) of the patients were scored having a high fall risk (3 or 4 risk scores).

**Conclusion:**

It was possible to develop a preliminary categorization of FRIDs basing on their adverse drug effect profile in SmPCs and frequency of use in older patients who had experienced at least one documented fall in a geriatric care unit. Even though more than half of the fall-experienced study participants had high fall risk scores on admission, their fall risk might have been underestimated as use of high fall risk medicines was common, even concomitant use. Further studies are needed to develop the FRID categorization and assess its impact on fall risk.

## Background

Falls constitute a leading preventable cause of geriatric injuries and hospitalization, prolonged recovery times and deaths [1, 2]. More than a third of older adults fall each year, meaning worldwide a high number of patients [3]. Fall-related injuries can be serious, occuring approximately in 10% of falls, and consequencing an urgent hospitalization [3, 4]. Furthermore, falls decrease self-sufficiency and quality of life among older adults, because falls can result in fear of falling, loss of confidence, mobility and ability to live independently [5].

Fall risk is a multifactorial problem associated with numerous intrinsic and extrinsic factors [6, 7]. Intrinsic factors are related to ageing, including age-related changes to the pharmacokinetic and pharmacodynamic effects of pharmacotherapies. Age-related pharmacokinetic changes are represented by drug absorption, distribution, metabolism and elimination route. The drug absorption is usually decreased due to changes in the gastrointestinal tract, e.g., reduced gastric secretion, loss of mucosal intestinal surface and decreased blood flow in splanchnic area. The drug distribution is affected by many factors, e.g., increase in fat compartment, decrease in total body water, muscle mass and serum albumin level. The distribution volume of water soluble drugs is decreased leading to their potential toxicity, while the distribution volume of lipid soluble drugs is increased leading to prolongation of elimination half-life and accumulation of the drug in fatty tissues. The drug metabolism is influenced by decreased hepatic blood flow and low activity of liver enzymes. The elimination route is limited due to reduction in renal clearance [7, 8]. Pharmacodynamic changes involve altered sensitivity to many pharmacological agents, increased sensitivity in psychotropic or cardiovascular drugs, in contrast with decreased capacity to respond to physiological challenges and side effects of the drug therapy, e.g., orthostatic hypotension [1, 9].

Extrinsic factors include fall risks related to the environment where individuals live, e.g., how medicines are taken at home [6, 10]. Prescribed medication is an important and potentially underappreciated contributor to falls [11, 12]. The older adults are susceptible to polypharmacy and higher risk of falls [13]. Polypharmacy can increase the risk of medication-related falls, especially when at least one established fall risk-increasing drug is part of the patient’s daily regimen [14]. A leading mechanism for increased risk can be sedation which slows reaction time, orthostatic hypotension, syncope, dizziness, drowziness, and blood pressure change or impaired balance [15]. Normally, long-acting medications pose a higher risk, but the metabolism and half-life of otherwise short-acting drugs can be also prolonged in older adults [16, 17]. Previous studies have presented various methods reducing fall risks in older adults, such as supplementation of vitamin D and calcium, cataract surgery, hip protectors, modification of hazard environment and adjustment of medications [18, 19]. In addition, stability of the human body is a crucial factor for reducing falls [20]. Recommended actions to improve stability include exercise programs focusing on balance and muscle strength [21]. There is a good evidence for reducing medication-induced falls. In psychotropics, the fall risk can be reduced by prospective management of adverse effects such as drowsiness, dizziness, slow reaction time and orthostatic hypotension [22, 23]. Drugs reducing blood pressure are associated with fall risk because of their hypotension effect. Thus, adjusting anti-hypertensive medication may reduce syncope and falls [24, 25]. The fall risk prevention with anticoagulants should focus on bleeding events such as a cerebral hemorrhage and associated falls [26]. Many drugs disturb vision and vestibular system, which exacerbate gait disturbance in older adults [27, 28]. In case of falls, reduced bone mineral density and osteoporosis can increase risk of fracture and more severe consequences [29].

Evidence on medication-related fall risks is not always integrated into multifactorial risk assessments which contribute to preventive programmes for reducing falls [30, 31]. Incorporating these risks could improve accuracy of existing tools routinely used in geriatric care units as many of the current workflows do not contain medication as a risk factor [32]. This study aimed to provide a better screen on falls in institutions by developing a preliminary categorization of fall-risk-increasing drugs (FRIDs) that could be added as a risk factor to the existing fall risk assessment tool routinely used in geriatric care units.

## Methods

### Patient data

The patient data used in this study were retrospectively derived from two different types of health care institutions, i.e., a hospital and a nursing home in Brno, Czech Republic. Medication data on all patients who had fallen at least once during their stay at a health care institution (hospital or nursing home) within a 2-year observational period were derived from patient records. The fall is defined by World Health Organization as an event which results in a person coming to rest inadvertently on the ground or floor or other lower level [31]. The falls during a stay at a health care institution were documented as undesirable events in a protocol, which contained information about circumstances of the fall such as a description of fall, exact time and place, and consequences, e.g., severe injuries, bone fracture or head injury, bruises or bleeding risk. Descriptive and demographic information on patients (sex, age, length of stay, MMSE score and medication use) were collected from the patient records. Inclusion criteria of the study participants were 1) age ≥ 60 years old, 2) evidence of at least one fall during their stay in a geriatric care unit, 3) evidence of fall documented by health care professionals (nurses, physicians). Exclusion criteria were 1) age < 60 years old, 2) absence of falls during their stay in a geriatric care unit, 3) no evidence of falls documented by health care professionals. The nursing home had less descriptive records of their residents, e.g., they missed a cognitive impairment scale.

### Developing categorization of medicines increasing fall risk (FRIDs)

The medication-related fall risk was determined by categorizing all medicines patients used according to each medicine’s fall risk. The criteria for this categorization was derived from the staturory summaries of product characteristics (SmPCs) approved by authorities as part of marketing authorization within European Union countries [33]. The SmPC information of each medicine was systematically reviewed by two researchers (JM, KV) to derive the fall risk. We applied information on a direct connection between the fall risk and adverse drug effects (ADEs) disturbing the patient’s balance as presented in SmPCs. This information was used to create an A, B, C medication-related fall risk categorization. Usually the mechanism leading to medication-related fall risk is one or more of the following adverse drug effects: sedation, orthostatic hypotension, syncope, dizziness, drowsiness, changes in blood pressure or impaired balance [34, 35]. All these adverse drug effects have a negative influence on a patient’s balance, which can then predispose the patient to falls [36].

Adverse drug effects posing fall risk are classified in the SmPC as very common (≥1/10), common (≥ 1/100), uncommon (≥ 1/1000), rare (≥ 1/10000) and very rare (< 1/10000) (Table 1). Medicines with at least one of the above-mentioned adverse drug effect frequencies of “very common” or “common” in the SmPC were categorized as high fall risk medicines (category A). Similarly, the medicines with an adverse drug effect frequency “uncommon” were categorized as moderate fall risk medicines (risk level B) and those with rare or very rare frequency as no fall risk medicines (risk level C). The high-risk category A medicines are considered as a predisposition factor to fall, because the occurrence of adverse drug effects connected to falls is documented by at least one patient per each 100 patients using the same active substance. The risk information provided in the SmPC of the brand product used by the patients involved in the study was validated by comparing the information with the SmPC information of another similar product on the market.

**Table 1:** Fall risk level criteria: categorization according to information derived from the summaries of product characteristics (SmPC) on the frequency of adverse drug effects (ADEs) contributing to fall risk (sedation, orthostatic hypotension, syncope, dizziness, drowsiness, changes in blood pressure or impaired balance)

Frequency of ADEs	Number of patients experiencing ADEs	Fall risk level
**Very common**	≥ 1/10	**A**
**Common**	≥ 1/100 to < 1/10	**A**
**Uncommon**	≥ 1/1000 to < 1/100	**B**
**Rare**	≥ 1/10000 to < 1/1000	**C**
**Very rare**	< 1/10000	**C**

### Frequency of use of high fall risk medicines in study participants with a fall history

The second phase of the study focused on counting the frequency of use of high fall risk medicines in study participants with a fall history in institutions. This phase made use of the existing routine practice at the time of the study of assessing and scoring all patients for fall risk by nurses in the routine admission procedure. Both institutions used the same set of 4 risk items other than medications to estimate fall risk (Table 2). The original Morse Fall Score [37] had been shortened to a time-saving procedure of 4 items suitable for clinical practice. Each fall risk item yielded 1 point and they assessed 1) history of falls, 2) mental condition, 3) physical condition, and 4) occurrence of dizziness or drowsiness in older patients. The estimation was based on patient interviews on admission or notes from patient records. Direct questions to the patient were dependent on the patient’s health condition. As this fall risk estimation did not consider patient’s medication as a contributor to the fall risk, the patient data on medication use were retrospectively collected from the patient records by the researcher (JM). All the medicines were classified into three risk categories (high, moderate and none) using the new categorization based on the fall risk information in the SmPCs. For the descriptive feasibility testing, the medication data of the patients in the hospital and nursing home were combined to provide the widest possible coverage of medicines used by older adults in institutions.

**Table 2:** The items included in the existing fall risk estimation routinely used in the two health care institutions participating in the study (a hospital and a nursing home)

Fall risk item	Responsible person	Confirmation	Points in the scale
Evidence of fall during a month prior the assessment	nurse	✓	1
Confusion, lack of risk perception, restlessness	nurse	✓	1
Impaired balance, gait disturbance	nurse	✓	1
Occurrence of dizziness and drowsiness	nurse	✓	1

### Statistical analysis

Descriptive statistics were used in both study phases to categorize active substances according to their fall risk, and to test the categorizations with a retrospective sample of patients from a secondary care hospital and a nursing home. All patients were grouped together for the analysis. Data were processed in Microsoft Excel, version 2016. A pivot table was created to analyze worksheet data. Descriptive statistics were used for patient data to count metric items such as frequencies, means, ranges, and standard deviations. The categorization of medicines according to their fall risk used ATC codes, specified in levels of anatomical main group, therapeutic subgroup, and pharmacological subgroup [38]. The commonly used active substances in the high fall risk category A were processed from the retrospective patient data as the frequency of use.

## Results

### Study participants

In total 188 fall-experienced older patients were retrospectively drawn from the patient registers of both institutions included in this study covering the 2-year period from January 2016 to December 2017 (Table 3). Of the patients, 128 (68%) were from the geriatric department of a secondary care hospital and 60 (32%) from a nursing home. Of them, 103 were females and 85 males with the mean age of 79 years (range: 60–97 years, standard deviation ±18.5). They used on average 9.3 medicaments (range: 2–17, standard deviation ±7.5) during a stay in a health care institution. The mean length of stay of the study patients in the secondary care hospital (*n* = 128) was 15.8 days (range: 1–56 days, standard deviation ±27.5). The most common causes of hospitalization were cardiovascular problems, and/or respiratory and urogenital tract infections. The half (52%) of the patients had at least a mild cognitive impairment (≤ 23 points), the mean score in the Mini Mental State Exam (MMSE) being 21.7 points (range: 5–30 points, standard deviation ±12.5). The stay for nursing home residents (*n* = 60) was permanent. Data about their cognitive function was not available. They suffered from many chronic diseases (cardiac, neurologic) and used chronic long-term medications.

**Table 3:** Characteristics of the participants from two health care institutions included in the study (*n* = 188, of which hospital inpatients, n = 128; nursing home residents, n = 60)

Characteristics		Hospital	Nursing home	Total
Sex n (%)	Male	67 (52)	18 (30)	85 (45)
	Female	61 (48)	42 (70)	103 (55)
Age, years, mean (range)		80 (60–97)	77 (63–95)	79 (60–97)
MMSE, mean (range)		21.7 (5–30)	unknown	–
Length of stay, days, mean (range)		15.8 (1–56)	permanent	–
Number of drugs in use, mean (range)		10.0 (3–17)	7.8 (2–15)	9.3 (2–17)

### The fall risk scores of study participants assessed on admission

Medications were not included in the routinely used assessment. Nurses had assessed and scored all patients (*n* = 188) for fall risk on admission using the original 4 risk items (score range 0–4). This fall risk assessment is a part of the regular procedure in each patient admission. More than half (53%, *n* = 100) of the patients were scored as having at least high fall risk (had 3 or 4 risk scores), the remaining patients (46%, *n* = 88) being scored with medium or lower risk (Table 4).

**Table 4:** Distribution of fall risk scores in fall-experienced patients (n = 188) using 4 fall risk items routinely assessed by nurses on admission (each item yielding 1 point, score range 0–4). Medications were not included in the assessement

Scores	Fall risk level	Number of patients	Proportion (%) of patients
**0**	No risk	5	3
**1**	Low risk	17	9
**2**	Medium risk	66	35
**3**	High risk	69	37
**4**	Full risk	31	16

### Categorization of medicines according to their fall risk by applying SmPCs and frequency of use

**Fig. 1:**
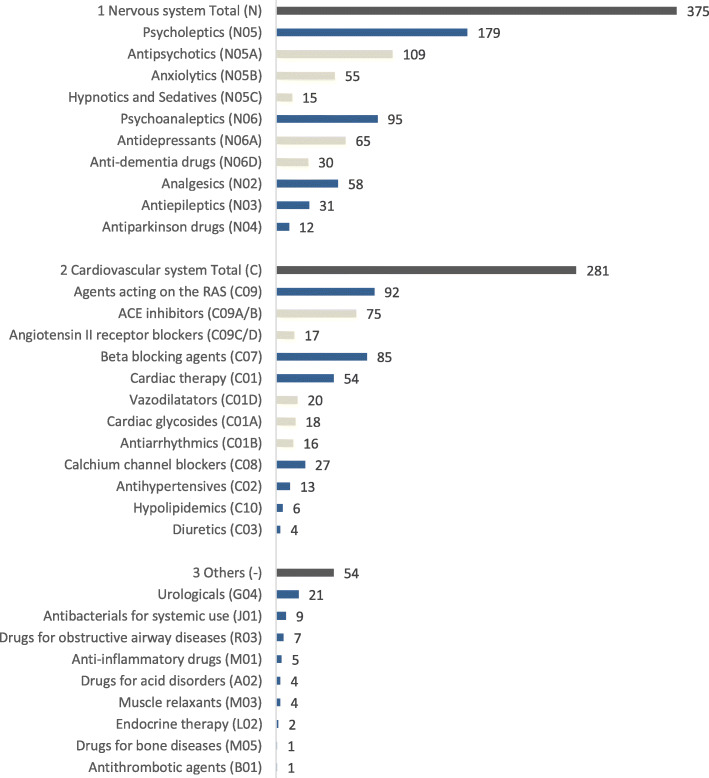
Categorization of high fall-risk Category A medicines (n = 710) according to therapeutic group and number of users. The medicines are organized according to the ATC Classification [38].. Anatomical main group (1st level, dark grey), Therapeutic subgroup (2nd level, blue), Pharmacological subgroup (3rd level, grey)

Fall-experienced patients (*n* = 188) used altogether 1748 medicaments. Of these medicaments, 710 (41%) belonged to the category of high fall risk (category A), 331 (19%) moderate risk (category B) and 707 (40%) no risk (category C). The mean number of high-risk medications (category A) in use per patient was 3.8 medications, minimum 0 (*n* = 2), maximum 8 (*n* = 4) and median being 3 (*n* = 46). The majority (93%) of high-risk category A medicines (*n* = 710) used by the patients were for the nervous system (53%, *n* = 375) and the cardiovascular system (40%, *n* = 281) (Fig. 1). The nervous system medicines in use were mainly psychotropic medications (73% of the medicines in use in this category), while analgesics (16%), antiepileptics (8%) and antiparkinson drugs (3%) represented a minority. Psychotropic medications (*n* = 274) consisted of 1) psycholeptics (*n* = 179, representing 65% of the medicines in this category) such as antipsychotics (*n* = 109, 40%), anxiolytics (*n* = 55, 20%), hypnotics and sedatives (*n* = 15, 5%), and 2) psychoanaleptics (*n* = 95, representing 35% of the medicines in this category) such as antidepressants (*n* = 65, 24%) and anti-dementia drugs (*n* = 30, 11%). The high fall risk cardiovacular medications (*n* = 281) included agents acting on the renin-angiotension system (RAS) (*n* = 92, 33% of the medicines in use in this category), beta blocking agents (*n* = 85, 30%), cardiac therapy (*n* = 54, 19%), calcium channel blockers (*n* = 27, 10%) and other therapeutic subgroups (*n* = 23, 8%).

Of the 216 active substances used by the patients, 102 (47%) were classified in the high-risk category (fall risk level A), 29 (13%) in the moderate risk category (fall risk level B) and 85 (40%) in no risk category (fall risk level C). The leading mechanisms of ADEs consequencing in high fall risk of most commonly used active ingredients by study participants are presented in Table 5. The most commonly used active ingredients in the high fall risk category A (*n* = 710) are presented in Table 6, included metoprolol (*n* = 49, beta blocking agents), perindopril (*n* = 38, ACE inhibitors), tiapride (*n* = 36, antipsychotics), quetiapine (*n* = 24, antipsychotics), tramadol (n = 24, opioids), citalopram (*n* = 23, antipressants), amlodipine (n = 23, calcium channel blockers), melperone (n = 23, antipsychotics), bromazepam (*n* = 20, anxiolytics), digoxin (*n* = 18, cardiac glycosides), and ramipril (n = 18, ACE inhibitors).

**Table 5:** The most commonly used fall risk increasing drugs (Category A) having very common (≥1/10) or common (≥1/100) frequency of ADEs connected to fall risk according to statutory summary of product characteristics (SmPCs)

Active ingredient (number of users)	Drug class (ATC)	Frequency of ADEs according to SmPCs
**Metoprolol (n = 49)**	Beta blocking agents (C07A)	Common: dizziness, bradycardia
**Perindopril (n = 38)**	ACE inhibitors (C09A)	Common: dizziness, orthostatic hypotension, syncope
**Tiapride (n = 36)**	Antipsychotics (N05A)	Common: dizziness, drowsiness
**Quetiapine (n = 24)**	Antipsychotics (N05A)	Very common: dizziness, drowsiness Common: orthostatic hypotension
**Tramadol (n = 24)**	Opioids (N02A)	Very common: dizziness Common: drowsiness
**Citalopram (n = 23)**	Antidepressants (N06A)	Very common: drowsiness Common: dizziness
**Amlodipine (n = 23)**	Calcium channel blockers (C08C)	Common: dizziness, drowsiness
**Melperon (n = 23)**	Antipsychotics (N05A)	Very common: sedation Common: dizziness
**Bromazepam (n = 20)**	Anxiolytics (N05B)	Common: drowsiness, falls^a^
**Digoxin (n = 18)**	Cardiac glycosides (C01A)	Common: bradycardia, dizziness
**Ramipril (n = 18)**	ACE inhibitors (C09A)	Common: dizziness, orthostatic hypotension, syncope

^a^unknown frequency of ADE

**Table 6:** The preliminary categorization of high fall risk medicines (Category A) basing on their adverse drug effect (ADE) profiles in statutory summaries of product characteristics (SmPCs) and frequency of use in older patients who had experienced at least one documented fall in a geriatric care unit (n = 188). The medicines are organized according to the ATC Classification [38]

High fall risk (A)	ATC Code	ATC classification	Users of the medicines n (%)
**Metoprolol**	C07AB02	Beta blocking agents	49 (26.1)
**Perindopril**	C09AA04	ACE inhibitors	38 (20.2)
**Tiapride**	N05AL03	Antipsychotics	36 (19.1)
**Quetiapine**	N05AH04	Antipsychotics	24 (12.8)
**Tramadol**	N02AX02	Opioids	24 (12.8)
**Citalopram**	N06AB04	Antidepressants	23 (12.2)
**Amlodipine**	C08CA01	Calcium channel blockers	23 (12.2)
**Melperone**	N05AD03	Antipsychotics	23 (12.2)
**Bromazepam**	N05BA08	Anxiolytics	20 (10.6)
**Ramipril**	C09AA04	ACE inhibitors	18 (9.6)
**Digoxin**	C01AA05	Cardiac glycosides	18 (9.6)
**Tramadol combination**	N02AJ13	Opioids	16 (8.5)
**Bisoprolol**	C07AB07	Beta blocking agents	16 (8.5)
**Zolpidem**	N05CF02	Hypnotics and Sedatives	15 (8.0)
**Donepezil**	N06DA02	Anti-dementia drugs	14 (7.4)
**Mirtazapine**	N06AX11	Antidepressants	14 (7.4)
**Amiodarone**	C01BD01	Antiarrhythmics	14 (7.4)
**Oxazepam**	N05BA04	Anxiolytics	14 (7.4)
**Tamsulosin**	G04CA02	Urologicals	14 (7.4)
**Memantine**	N06DX01	Anti-dementia drugs	12 (6.4)
**Diazepam**	N05BA01	Anxiolytics	11 (5.9)
**Gabapentin**	N03AX12	Antiepileptics	11 (5.9)
**Trazodone**	N06AX05	Antidepressants	10 (5.3)
**Isosorbide mononitrate**	C01DA14	Vasodilatators	10 (5.3)
**Clonazepam**	N03AE01	Antiepileptics	10 (5.3)
**Codein combination**	N02AJ06	Opioids	9 (4.8)
**Risperidone**	N05AX08	Antipsychotics	8 (4.3)
**Losartan**	C09CA01	AT II receptor blockers	8 (4.3)
**Sertraline**	N06AB06	Antidepressants	7 (3.7)
**Levodopa**	N04BA01	Dopaminergic agents	7 (3.7)
**Aminophylline**	R03DA05	Drugs for obstructive airway diseases	7 (3.7)
**Carvedilol**	C07AG02	Beta blocking agents	7 (3.7)
**Fentanyl**	N02AB03	Opioids	6 (3.2)
**Rosuvastatin**	C10AA07	Lipid modifying agents	6 (3.2)
**Betaxolol**	C07AB05	Beta blocking agents	6 (3.2)
**Haloperidol**	N05AD01	Antipsychotics	6 (3.2)
**Levomepromazine**	N05AA02	Antipsychotics	6 (3.2)
**Pregabalin**	N03AX16	Antiepileptics	6 (3.2)
**Nitrofurantoin**	J01XE01	Antibacterials for systemic use	6 (3.2)
**Urapidil**	C02CA06	Antiadrenergic agents	5 (2.7)
**Rilmenidine**	C02AC06	Antiadrenergic agents	5 (2.7)
**Nebivolol**	C07AB12	Beta blocking agents	5 (2.7)
**Escitalopram**	N06AB10	Antidepressants	5 (2.7)
**Telmisartan**	C09CA07	AT II receptor blockers	5 (2.7)
**Midazolam**	N05CD08	Hypnotics and Sedatives	5 (2.7)
**Alprazolam**	N05BA12	Anxiolytics	5 (2.7)
**Perindopril/Indapamide**	C09BA04	ACE inhibitors combination	5 (2.7)
**Verapamil**	C08DA01	Calcium channel blockers	4 (2.1)
**Amiloride/Hydrochlorthiazide**	C03EA01	Diuretics	4 (2.1)
**Rivastigmine**	N06DA03	Anti-dementia drugs	4 (2.1)
**Olanzapine**	N05AH03	Antipsychotics	4 (2.1)
**Dutasteride/Tamsulosin**	G04CA52	Urologicals	4 (2.1)
**Trimetazidine**	C01EB15	Other cardiac preparations	4 (2.1)
**Fosinopril**	C09AA09	ACE inhibitors	3 (1.6)
**Isosorbide dinitrate**	C01DA08	Vasodilatators	3 (1.6)
**Aceclofenac**	M01AB16	Anti-inflammatory drugs	3 (1.6)
**Captopril**	C09AA01	ACE inhibitors	3 (1.6)
**Baclofen**	M03BX01	Muscle relaxants	3 (1.6)
**Lansoprazole**	A02BC03	Drug for peptic ulcer and reflux	3 (1.6)
**Cefuroxime**	J01DC02	Antibacterials for systemic use	3 (1.6)
**Levetiracetam**	N03AX14	Antiepileptics	2 (1.1)
**Propafenone**	C01BC03	Antiarrhythmics	2 (1.1)
**Carbamazepine**	N03AF01	Antiepileptics	2 (1.1)
**Diclofenac**	M01AB05	Anti-inflammatory drugs	2 (1.1)
**Solifenacin/Tamsulosin**	G04CA53	Urologicals	2 (1.1)
**Oxycodone**	N02AA05	Opioids	2 (1.1)
**Trandolapril**	C09AA10	ACE inhibitors	2 (1.1)
**Levodopa/Carbidopa**	N04BA02	Dopaminergic agents	2 (1.1)
**Perindopril/Amlodipine/Indapamide**	C09BX01	ACE inhibitors combination	2 (1.1)
**Bicalutamide**	L02BB03	Hormone antagonists	2 (1.1)
**Glyceryl trinitrate**	C01DA02	Vasodilatators	2 (1.1)
**Fluoxetine**	N06AB03	Antidepressants	1 (0.5)
**Dosulepin**	N06AA16	Antidepressants	1 (0.5)
**Mianserin**	N06AX03	Antidepressants	1 (0.5)
**Candesartan**	C09CA06	AT II receptor blockers	1 (0.5)
**Venlafaxine**	N06AX16	Antidepressants	1 (0.5)
**Telmisartan/Hydrochlorothiazide**	C09DA07	AT II receptor blockers combination	1 (0.5)
**Tizanidine**	M03BX02	Muscle relaxants	1 (0.5)
**Lisinopril**	C09AA03	ACE inhibitors	1 (0.5)
**Famotidine**	A02BA03	Drug for peptic ulcer and reflux	1 (0.5)
**Quinapril/Hydrochlorothiazide**	C09BA06	ACE inhibitors combination	1 (0.5)
**Naftidrofuryl**	C04AX21	Peripheral vasodilatators	1 (0.5)
**Celiprolol**	C07AB08	Beta blocking agents	1 (0.5)
**Paroxetine**	N06AB05	Antidepressants	1 (0.5)
**Methyldopa**	C02AB01	Antiadrenergic agents	1 (0.5)
**Tianeptine**	N06AX14	Antidepressants	1 (0.5)
**Doxazosin**	C02CA04	Antiadrenergic agents	1 (0.5)
**Ticagrelor**	B01AC24	Antithrombotic agents	1 (0.5)
**Losartan/Hydrochlorothiazide**	C09DA01	ACE inhibitors combination	1 (0.5)
**Codeine**	R05DA04	Cough suppressants	1 (0.5)
**Acebutolol**	C07AB04	Beta blocking agents	1 (0.5)
**Mirabegron**	G04BD12	Urologicals	1 (0.5)
**Ibandronic acid**	M05BA06	Drugs affecting bone	1 (0.5)
**Levodopa/Benserazide**	N04BA02	Dopaminergic agents	1 (0.5)
**Selegiline**	N04BD01	Dopaminergic agents	1 (0.5)
**Moxonidine**	C02AC05	Antiadrenergic agents	1 (0.5)
**Irbesartan**	C09CA04	ACE inhibitors	1 (0.5)
**Perindopril/Amlodipine**	C09BB04	ACE inhibitors combination	1 (0.5)
**Cilazapril**	C09AA08	ACE inhibitors	1 (0.5)
**Fluphenazine**	N05AB02	Antipsychotics	1 (0.5)
**Ropinirole**	N04BC04	Dopaminergic agents	1 (0.5)
**Chlorprothixene**	N05AF03	Antipsychotics	1 (0.5)

## Discussion

This study developed a preliminary categorization to identify medicines that may increase a fall risk in older adults in institutions, by using evidence derived from statutory medicines information and retrospective patient records. Our results indicate that numerous medicines have such an ADE profile that can lead to an increased fall risk and that the use of these high fall-risk medicines, even concomitant use of several active ingredients, is common in older adults during a hospital stay or while living in a nursing home. These findings suggest incorporating a medication-related fall risk indicator in a routine fall risk assessment on admission to geriatric care units. In long-term care units such as nursing homes, it may be useful to repeat the fall risk assessment regularly, e.g., at least once a year.

The high-fall risk categorization presented in this study considered also patient data which gives an idea of ​​the prevalence of clinical use of different medicines. By this way it was possible to identify a few widely used medicines with a high fall risk. The top three clearly most commonly used high fall risk active substances were metoprolol (beta blocking agent), perindopril (ACE inhibitor) and tiapride (antipsychotic agent). Of the top ten active ingredients, 6/10 were psychotropics (3 of them antipsychotics) and 4/10 cardiovascular agents. Optimizing the use of these few medicines in terms of prospectively managing fall risk could make a remarkable change in the incidence of actual falls in geriatric care units.

Our findings concerning the “ranking “of drugs with a high fall risk are in line with previous research. The association with falls has been consistently reported for psychotropic and cardiovascular medicines [39–45]. The recent systematic reviews and meta-analyses (Seppala [43] and de Vries [44]) confirmed the association between certain drug classes and fall risk from psychotropics, i.e., antidepressants (OR = 1.57), antipsychotics (OR = 1.54), and benzodiazepines (OR = 1.42), and cardiovascular drugs, i.e., cardiac glycosides (OR = 1.6), antiarrhythmics (OR = 1.27), vazodilatators (OR = 1.03), and ACE inhibitors (OR = 1.03). The increased fall risk is also evidenced in patients using opioids (OR = 1.61), antiepileptics (OR = 1.55), anti-Parkinson drugs (OR = 1.54), and NSAIDs (OR = 1.09) [46, 47]. Furthermore, the previous study from Czech Republic (Maly et al. [48]) classified drugs that affected to the nervous system (antipsychotics, antidepressants, analgesics) and to the cardiovascular system (diuretics, beta blocking agents, agents acting on the renin-angiotensin system) as the most frequently used fall risk-increasing drugs in hospitals. Thus, many studies have reached similar conclusions, although the fall-increasing medicines have been presented slightly in different order. The identification of increased risk of falls in different drug classes might be a crucial risk factor for falls. The European Geriatric Medicine Society and Finnish Expert Group on Fall-Risk-Increasing Drugs (FRIDs) have concluded that the knowledge about the risk of falls associated with therapeutic classes and individual medications can help in fall prevention [49].

According to our findings, the fall risk assessment tool presented in this study could be helpful to prevent medication-related falls and increase quality of geriatric care in health care institutions. This is supported by the findings of previous studies that have reported the medication use as a remarkable but modifiable fall risk factor [48, 50]. Therefore, it is reasonable to implement a strategy to avoid use of fall risk-increasing drugs in the routine practice in geriatric care units. Nevertheless, many of developed fall risk assessment tools do not consider medication as a risk factor included in the tool. A review of twenty fall risk assessment tools found that only seven of the tools consider medication use as a risk factor [32]. More recent tools include medication use in a fall risk assessment, e.g., The Johns Hopkins Fall Risk Assessment Tool: it ranks opiates, anticonvulsants, anti-hypertensives, diuretics, hypnotics, laxatives, sedatives, and psychotropics among high fall risk drugs [51].The Johns Hopkins Fall Risk Assessment Tool is well-structured and validated, but the total number of risk points to evaluate is high (maximum 28). This makes it more time consuming for health care providers to assess the fall risk than by using a tool with fewer risk points, such as the five items suggested to be used in our updated fall risk assessment tool. Our tool is also unique in the way that the list of fall risk medicines was created basing on statutory medicines information presented in the summaries of product characteristics approved by European Union (EU) authorities.

This study indicates that the current practice of fall risk estimation in geriatric care units without including medications underestimates the actual fall risk. More than half (53%, *n* = 100) of the patients included in our study scored at least a high fall risk, but the remaining 46% (*n* = 88) of these fall-experienced patients scored a medium risk (35%), a low risk (9%) or no risk (3%). The addition of a new item scoring the exposure to the high fall risk medication could refine the prospective risk assessments. This new risk item represented by high fall risk medicines (FRIDs) should be considered in each patient admission or medication change and periodically assessed in the existing fall risk assessment tool by health care experts.

Fine-tuning the fall risk scoring system requires further research and comparisons with the contents of other existing tools. Further research should compare how much adding high risk medicines to the fall risk assessment tool changes the risk score and makes the risk score more accurate in terms of predicting and preventing actual falls. It also might be useful to assess whether concomitant use of more than one FRID medicines will elevate the fall risk. Determining this in future studies may be necessary because the concomitant use of several drugs that increase the risk of falls was common in our data (on average 3.8 high risk medicaments per patient). In addition to this kind of risk verification research, more generalizable results are needed by testing the fall risk assessment tool with a larger number of geriatric patients from a larger number of hospital wards and nursing homes. These studies should be based on prospective patient data. Furthermore, the fall risk profiles and patterns of the high fall risk medicines should be further investigated in older adults in hospital and home care settings.

### Study limitations

When interpreting the results of this study it is important to keep in mind that the study does not cover the complete spectrum of medicines used by older adults, only the medicines used by the 188 study patients. Furthermore, the retrospective patient and medication data from the patient records may perform as a source of bias. The data related to the nursing home residents were not documented in such detail compared to that of hospital patients. The differences in the detailedness of documentation concern e.g., information on cognitive impairment or changes made to medications of individual patients. Documentation may have missed minor falls which were not reported to the staff by the patients.

## Conclusion

It was possible to develop a preliminary categorization of FRIDs basing on their adverse drug effect profile in SmPCs and frequency of use in older patients who had experienced at least one documented fall in a geriatric care unit. Even though more than half of the fall-experienced older adults had at least high fall risk scores on admission according to the fall risk items routinely assessed, their fall risk might have been underestimated as use of high fall risk medicaments was common, even concomitant use. Further studies are needed to develop the FRID categorization and assess its impact on fall risk.

## Data Availability

The datasets derived from summaries of product characteristics during the current study are available from the corresponding author on reasonable request. The datasets generated and analysed during this study that were derived from retrospective patient records are not publicly available due to regulations on secondary use of patient data and subsequent agreements with the institutions to perform the study.

## Abbreviations

ADE: Adverse Drug Effect
ATC Classification: Anatomical Therapeutic Chemical Classification
EU: European Union
FRID: Fall Risk Increasing Drug
MMSE: Mini Mental State Exam
OR: Odds Ratio
SmPC: Summary of Product Characteristic
